# Elevated Neurokinin-1 Receptor Expression in Uterine Products of Conception Is Associated With First Trimester Miscarriages

**DOI:** 10.3389/fphys.2020.554766

**Published:** 2020-12-18

**Authors:** Ahmad Alwazzan, Riffat Mehboob, Amber Hassan, Shahida Perveen, Sadaf, Syed Amir Gilani, Fridoon Jawad Ahmad, Imrana Tanvir, Masroor Elahi Babar, Muhammad Akram Tariq, Gibran Ali, Shehla Javed Akram, Rizwan Ullah Khan, Javed Akram

**Affiliations:** ^1^Division of Gynecology Oncology, Faculty of Medicine, King Abdulaziz University, Jeddah, Saudi Arabia; ^2^SISSA, International School for Advanced Studies, Trieste, Italy; ^3^Faculty of Allied Health Sciences, The University of Lahore, Lahore, Pakistan; ^4^Department of Pathology, Continental Medical College, Lahore, Pakistan; ^5^Center for Research in Molecular Medicine, The University of Lahore, Lahore, Pakistan; ^6^Department of Physiology and Cell Biology, University of Health Sciences, Lahore, Pakistan; ^7^Institute for Regenerative Medicine, University of Health Sciences, Lahore, Pakistan; ^8^Department of Pathology, King Abdulaziz University, Jeddah, Saudia Arabia; ^9^The University of Agriculture, Dera Ismail Khan, Pakistan; ^10^Department of Biology, Department of Postgraduate College, Township, Lahore, Pakistan; ^11^Akram Medical Complex, Lahore, Pakistan; ^12^Department of Pathology, Prince Faisal Cancer Center, King Fahad Specialist Hospital, Buraidah, Saudi Arabia

**Keywords:** miscarriages, abortions, neurokinin 1 receptor, substance P, NK-1R antagonists

## Abstract

**Background:**

Miscarriage is a common complication of early pregnancy, mostly occurring in the first trimester. However, the etiological factors and prognostic and diagnostic biomarkers are not well known. Neurokinin-1 receptor (NK-1R) is a receptor of tachykinin peptide substance P (SP) and has a role in various pathological conditions, cancers, but its association with miscarriages and significance as a clinicopathological parameter are not studied. Accordingly, the present study aimed to clarify the localization and expression for NK-1R in human retained products of conception (POC). The role of NK-1R is not known in miscarriages.

**Materials and Methods:**

NK-1R expression was assessed in POC and normal placental tissues by immunohistochemistry. Three- to four-micrometer-thin sections of formalin-fixed paraffin-embedded tissues were used for this purpose. Tissues were processed and then immunohistochemically stained with NK-1R antibody. Brain tissue was used as control for antibody. Protein expression was evaluated using the nuclear labeling index (%). Tissues were counterstained with 3,3′-diaminobenzidine (DAB), and microscopy was performed at 10×, 20×, and 40× magnifications.

**Results:**

Ten human POC tissues and 10 normal placental tissues were studied by immunohistochemistry to demonstrate the localization of NK-1R. The expression of NK-1R protein was high in all the cases of both groups. NK-1R expression showed no notable differences among different cases of miscarriages as well as normal deliveries at full term regardless of the mother’s age and gestational age at which the event occurred. Statistically, no difference was found in both groups, which is in agreement with our hypothesis and previous findings.

**Conclusion:**

The expression of NK-1R was similar in all the cases, and it was intense. It shows that dysregulation of NK-1R along with its ligand SP might be involved in miscarriages and also involved in normal delivery. Our results provide fundamental data regarding this anti-NK-1R strategy. Thus, the present study recommends that SP/NK-1R system might, therefore, be considered as an emerging and promising diagnostic and therapeutic strategy against miscarriages. Hence, we report for the first time the expression and localization of NK-1R in POC. We suggest NK-1R antagonist in addition to the immunoglobulins and human chorionic gonadotropin to diagnose and treat spontaneous miscarriages.

## Introduction

Miscarriages or spontaneous abortion in the initial stage of pregnancy is a common problem in the first trimester of pregnancy (Strommen et al., 2017). Many factors are involved in it, and it is a complex phenomenon [27]. Approximately 15% of pregnant females miscarry spontaneously without any known cause (Shetty and Narasimha, 2016). Miscarriages are further divided into incomplete, complete, missed, and anembryonic miscarriages (Kling et al., 2018). Fifty percent of causes are still unknown, and no indicators have been identified in them. Tests to diagnose these cases include an ultrasound scanning and measurement of human chorionic gonadotropin (hCG). In this way, the patients at risk are identified. However, there is a dire need to explore potentially efficient biomarkers for the diagnosis of at-risk patients earlier than the onset of clinical symptoms or the unfortunate occurrence of event (Monteiro et al., 2020). It will not only provide a diagnostic strategy but also pave way for therapeutic interventions to manage such cases. For this purpose, neurokinin-1 receptor (NK-1R) is explored in the current study to evaluate its expression and localization in the products of conception (POC) tissues after a miscarriage.

NK-1 is a receptor of substance P (SP) protein, which is one of the peptides released from sensory nerves, and causes the enhancement of cellular excitability in several human tumor cells (Javid et al., 2020). There are two distinct conformational isoforms of NK-1R: a full-length NK-1R (NK-1RF) isoform and a truncated NK-1R (NK-1RT) isoform. The NK-1RT isoform lacks the terminal cytoplasmic 96-aa residues (Chernova et al., 2009). Both of these isoforms have the same binding affinity for SP but different affinities for NKA. The NK-1R has a relatively long 5′ untranslated region compared to the other tachykinin (TK) receptors, which is preceded by a single TATAAA sequence (Hershey and Krause, 1990).

The neurokinins are a class of peptide signaling molecules that mediate a range of central and peripheral functions including pain processing, gastrointestinal function, and stress responses like hematopoiesis (Chow et al., 2020), wound healing (Suvas, 2017), increased vascular permeability, neurogenic inflammation, leukocyte infiltration, cell survival, and anxiety (Schank, 2020). It is also involved in carcinogenesis as reported in many studies and leads to metastasis (Munoz et al., 2014, 2015; Mehboob et al., 2015; Javid et al., 2019). SP has a variety of physiological functions in humans, particularly, nervous, immune, and cardiovascular systems (Vilisaar and Arsenescu, 2016). Mainly, SP is a brain–gut hormone, and its receptor is present in brain regions but also in peripheral tissues. SP binds to NK-1R to carry out transmission of pain, secretions from the paracrine and endocrine system, vasodilation, and proliferation of cells (Garcia-Recio and Gascón, 2015; Amiri and Hashemy, 2019). It is a neuromodulator, neurotransmitter, as well as a neurohormone.

TKs possess a widespread distribution in central and peripheral nervous system that is undoubtedly a major source of these peptides. However, TKs also have a limited distribution in non-neural structures represented by the irregular and sparse localizations in which they display known and unknown functions. In neuronal cells, the active TKs act as neurotransmitters/neuromodulators; in non-neuronal cells, as autocrine, paracrine, and endocrine regulators.

SP brings most of its cellular activities after binding to the G protein-coupled receptor (GPCR) NK-1R. NK-1R has 407 amino acids and is encoded by TACR-1 gene. It is located on the cell surface (Helke et al., 1990). SP binds to heterotrimeric GPCR, with a preference for Gs and Gq like all TK receptors. Binding of receptor to Gs stimulates adenylyl cyclase and cyclic AMP production, whereas binding of receptor to Gq stimulates the phosphatidylinositol cascade (Nakanishi, 1991). Upon binding of SP to NK-1R, a signal transduction cascade is initiated by internalization of SP–NK-1R complex (Quartara and Maggi, 1997) that activates phospholipase C (PLC).

NK-1R, stimulated directly, may cause vasodilation of feto–placental blood vessels (Lowry and Woods, 2018). In a study, SP has been found to be expressed in trophoblastic cell tissues, but not observed in blood vessels of the fetus. Messenger RNA (mRNA) of NK-1R has been reported in placental tissue (Munoz et al., 2010; Obstetrics Subgroup et al., 2016).

In our previous study, we have postulated a theory based on evidence that SP/NK-1R expression may be variable in different developmental phases of humans whereby its expression is lower in normal fetus and elevated soon after birth and in infants. However, it decreased in adults, while if it is *vice versa*, it may cause sudden unexpected deaths (Mehboob, 2017). Accordingly, the present study aimed to clarify the localization and expression of NK-1R in human retained POC. The role of NK-1R is not known in retained POC. Identification of the cause is challenging, and there are no effective measures available for treatment.

## Materials and Methods

### Tissue Samples

The present study was approved by the Ethical Review Committee of The University of Lahore. Ten samples of POCs after different stages of spontaneous miscarriages and 10 normal placental tissues after full-term successful delivery were obtained from the Gynaecology Department, The University of Lahore Teaching Hospital, Lahore. The ages of the females ranged from 20 to 40 years with a mean age of 29 ± 11.5 years in females whose POCs were used in this study. The mean age of females who delivered successfully was 31 ± 7.5 years, and their normal placental tissue was obtained. The gestational age ranged from 1 and 12 weeks, and the mean weight of the fragments was 295 g. The mean gestational age of females with full-term normal delivery was 37 ± 1.3 weeks.

### Immunohistochemical Staining for Neurokinin-1 Receptor

Human POC tissues and normal placental tissues were studied by immunohistochemistry to demonstrate the localization of NK-1R. Tissues of POCs were fixed in 10% formalin and then embedded in paraffin. Four-micrometer-thick serial sections were cut and processed for immunohistochemical detection of NK-1R. Antigen retrieval was done in 10 mM citrate buffer (pH 6.0), followed by peroxidase blocking (Dako Cytomation A/S) on the sections, and then heated at 100°C for 60 min. NK-1R antibody (Abbott; 1:1,000) was applied and incubated for 30 min at 37°C and incubated in Universal Secondary Antibody (Roche Diagnostics KK) for 20 min at 37°C. DAB Map detection kit (Roche Diagnostics KK) was used for visualization. Nuclei were counterstained using a hematoxylin counterstain reagent (Roche Diagnostics KK).

We studied representative samples of POCs and placental tissues. The evaluation of all slides was done by two independent pathologists. In each slide, high-power microscopic fields were evaluated using a 10×, 20×, and 40× magnification. The presence or absence of staining and the intensity of the immunoreactivity were noted, as well as the number and type of cells. Intensity of staining was observed as a brown staining. The localization of staining, whether or not the staining was localized in the nucleus, cytoplasm, or plasma membrane, was also observed. The results were recorded as positive when they showed cellular and/or plasma membrane staining ranging from moderate to strong in more than 10% of the cells. By consensus among the pathologists, the intensity of the immunoreactive cells was scored as follows: when less than 10% of the total cells were stained, the number of immunoreactive cells was considered low (+1); it was considered moderate when 10–40% were stained (+2) and high when more than 40% were stained (+3) (Shima et al., 2010). The specimens were examined and photographed at 10×, 20×, and 40× magnification utilizing a digital microscope camera (Olympus AX80 DP21; Olympus, Tokyo, Japan) interfaced with a computer. All protein levels were evaluated using the nuclear labeling index (%), recorded as the percentage of positively stained nuclei in 100 cells in the selected area.

## Results

### Neurokinin-1 Receptor Protein Expression in Products of Conception and Placental Tissue Determined by Immunohistochemical Staining

**FIGURE 1:**
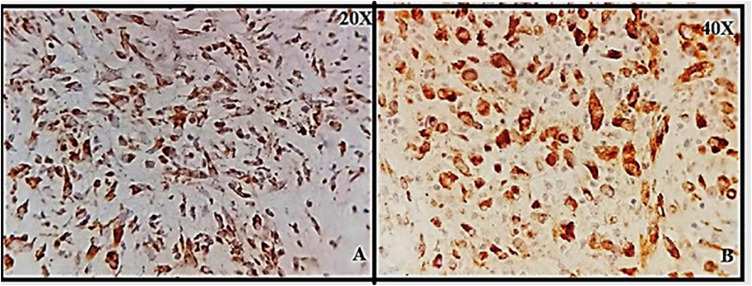
**(A,B)** Granular cytoplasmic positive staining in brain glial cells; positive control for neurokinin-1 receptor (NK-1R) at 20 and 40×.

**FIGURE 2:**
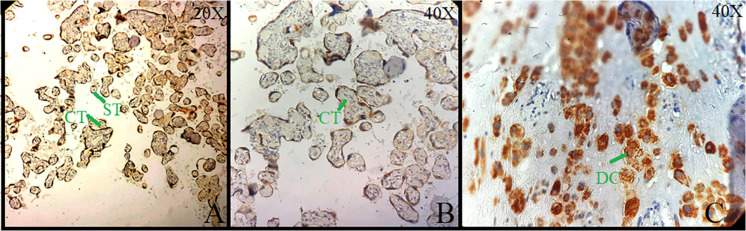
Immunolocalization of neurokinin-1 receptor (NK-1R) in normal placental tissue. **(A)** Strong cytoplasmic staining in cytotrophoblast (CT), syncytiotrophoblast (ST) at 20×. **(B)** Cytoplasmic staining in CT at 40×. **(C)** Sheets of decidual cells (DCs) with positive membrane and cytoplasmic NK-1R at 40×.

**FIGURE 3:**
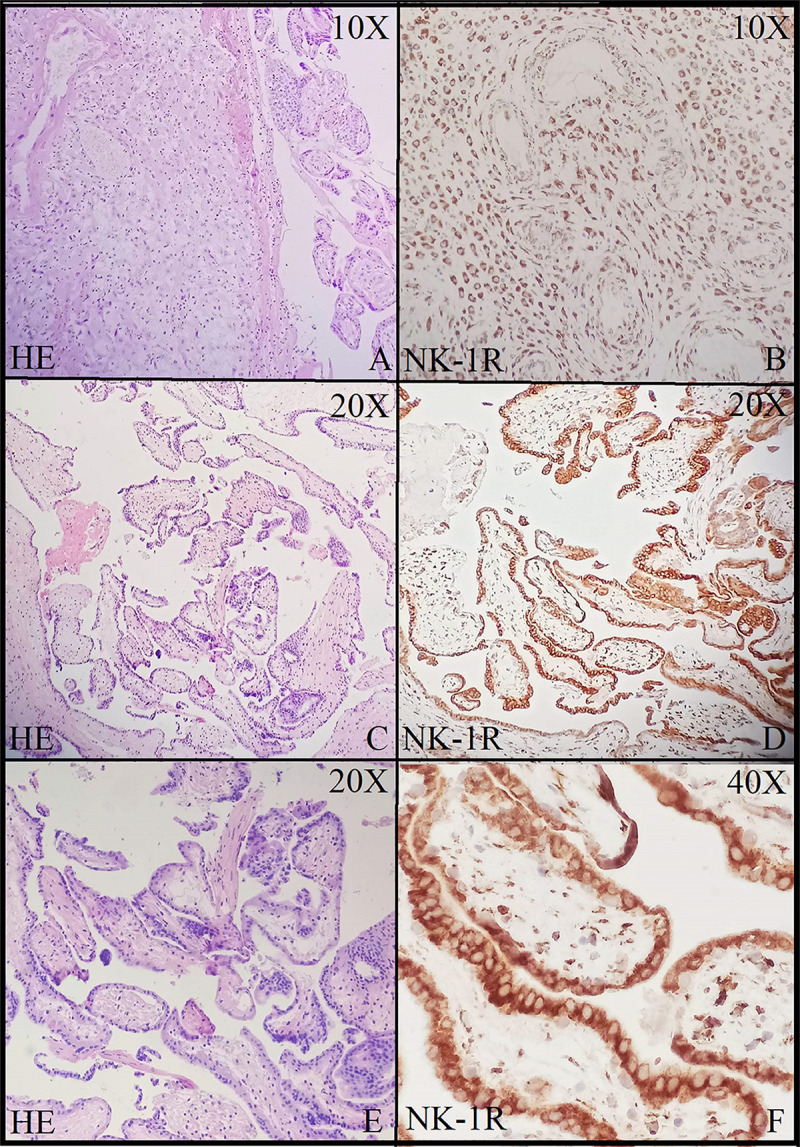
Neurokinin-1 receptor (NK-1R) immunohistochemical staining in products of conception showing intense staining and positive expression in all the cells at 10× **(A,B)**, 20× **(C,D)**, and 40× **(E,F)**.

Brain tissue was taken as a positive control for NK-1R, and it showed intense staining of +3 in all the cells (Figures 1A,B). The expression of NK-1R protein was high in all the cases of POCs when evaluated in all the stages of miscarriages. NK-1R expression showed no notable differences among different cases of miscarriages regardless of the age of females and the gestational age at which the event occurred. The NK-1R was widely distributed in the fetal membranes and placental tissues. The staining was high in epithelial cells of decidua, trophoblast of the fetal membranes, and chorionic villi (cytotrophoblast and syncytiotrophoblast). NK-1R was expressed in all the cells of POCs, whether maternal or fetal, in their epithelial membranes and nuclei. We determined the immunohistochemical staining of NK-1R, which is expressed mainly in the brain (Figures 1A,B). NK-1R levels were high and showed intense +3 expression in normal placental tissue (Figures 2A–C) as well as POCs (Figures 3A–F), with not much difference. Furthermore, we evaluated the nuclear labeling index of NK-1R using semi-serial sections. The NK-1R also showed no difference when comparing the cases within the POC group or normal placental group.

### Statistical Analysis

Median and interquartile range (IQR) were calculated for patient’s age and gestational age. Mann–Whitney *U*-test was applied to check the significant difference between NK-1R staining and groups. *P* < 0.05 was considered significant.

The median age of females whose retained POC were obtained was 29 ±11.5 years, and gestational age was 7 ± 3.3 weeks. The median age of females whose normal placenta was obtained was 31 ± 7.5 years, and gestational age was 37 ± 1.3 weeks. There was insignificant association between NK-1R staining and groups (Table 1).

**TABLE 1:** Mann–Whitney *U*-test on neurokinin-1 receptor (NK-1R) staining and groups.

Tissue	Female’s age (years) (median + IQR)	Gestational age in weeks (median + IQR)	NK-1R staining (median + IQR)	*P*-value
POCs	29 ± 11.5	7 ± 3.3	3.00 ± 0.0	0.7391
Normal placenta	31 ± 7.5	37 ± 1.3	3.00 ± 0.0	

## Discussion

This study reports for the first time about the detailed expression of NK-1R in POC at an early gestational age in the first trimester. Secondly, SP/NK-1 receptor system dysregulation may be involved in the pathology of pregnancy, such as abortion (Vilisaar and Arsenescu, 2016; Amiri and Hashemy, 2019), preeclampsia (Shima et al., 2010), and preterm birth. We describe here the immunolocalization of NK-1R in human POC, and we provide evidence that NK-1R is expressed in the nucleus. All these observations suggest that the NK-1R and SP have a role in the physiology of pregnancy. In our view, the demonstration that NK-1R in uterine products is associated with first trimester miscarriages has an important functional implication. NK-1R has a role in female reproduction. It has been known that mRNA for preprotachykinin-A, which encodes SP, is expressed in bovine corpus luteum (CL) of an early developmental stage; CL with a retained oocyte shows that the muscular apparatus of the preovulatory follicle has a role in oocyte expulsion and that the follicle wall contraction was missing in the mutant group. This may suggest luteinized unruptured follicle syndrome in humans (Patak et al., 2000).

There are several reports on the involvement of TKs in reproduction (Clement et al., 2009). All the TKs and their receptors are found to be expressed in the uterus of super-ovulated and unfertilized mice and may play a role in both male (Pintado et al., 2003) and female reproductive systems (Schmidt et al., 2003). There is a significant upregulation of NK-1R protein at full-term fetus and newborn infant with a peak at day 1, and it downregulates at day 8, which indicates that NK-1R may be involved in the mechanisms modulating the processes during labor and after birth. SP-IR has an opposite correlation with NK-1R protein expression in pregnancy and uterus after birth (Lavezzi et al., 2015). It correlates with our own previous study (Mehboob, 2017), in which it was proposed, based on our experimental work on sudden infant death syndrome, sudden fetal death victims, and controls (Mehboob et al., 2017), that SP expression is normally low in healthy fetuses but higher in neonates as compared to controls, and if *vice versa*, it may lead to sudden death. Furthermore, it was proposed that the expression of SP is variable, depending on the developmental stage. It is lower in adults and, if increased, it may lead to death (Mehboob, 2017). This study provided further evidence to strengthen our previous hypothesis. The possible reason is that SP is involved in cardiorespiratory control centers of the brain and has many important functions (Munoz et al., 2013; Mehboob et al., 2014). It regulates and controls the sleep–wake cycle, respiratory rhythm as well. If there is any disturbance in its regulation, it may cause fatal outcomes including death. NK-1R is a receptor of SP, and all the functions of SP are only initiated, regulated, and modulated once SP binds with NK-1R. Here, in this study, we found an increased NK-1R expression, which shows that if SP is increased in the initial stage of pregnancy, it may cause spontaneous abortion or miscarriage; if at any fetal stage it may initiate the respiratory mechanisms, which is injurious, as the lungs have not yet started functioning and gaseous exchange is *via* feto–placental route. At full term, there is a need to expel the fetus, there may be a rise in SP expression, leading to birth of the baby, and the same SP is required for respiration through the lungs after birth. Our current result showing a strong NK-1R expression in placental tissue supports this point. Ebner and Singewald (2006) and Munoz et al. (2010) observed the expression of SP and NK-1R in all the cells of placenta, which is in line with our current and previous findings, e.g., SP levels are raised at term fetus, near birth, and soon after. We may speculate that SP is regulating the pregnancy and controlling the respiratory mechanisms, delivery, and cardiorespiratory controls.

The role of SP in stress and anxiety is already established (Zieglgansberger, 2019). It is released more in such conditions as well as other nociceptive stimuli including pain (Fest et al., 2006). Stress-induced abortions may induce a rise in decidual tumor necrosis factor (TNF)-alpha and cause neurogenic inflammation. TNF-alpha is a possible stimulator for miscarriage (Joachim et al., 2001; Mowa et al., 2003). We already know that stress, pain, anxiety may lead to spontaneous miscarriage or preterm delivery as well. It shows that the possible mechanism may be *via* SP/NK-1R system. A study reported similar findings—that SP expression was elevated in full-term fetus and in newborn and may have played a role in cervical ripening and labor (Qian et al., 2018).

Pregnancy is a unique physiological process. During the early developmental stages, an immune rejection caused by fetal antigens is inhibited by the mother (Patak et al., 2000). The immune system has a very important role in pregnancy. During implantation, the endometrium makes the maternal–fetal connection and recruits T regulatory cells to the site, which is the local immune response (Wang et al., 2019). If there is any insufficiency in this process, it may cause spontaneous abortion (Mehboob et al., 2020). It may be speculated that increased immune reactions or hypersensitivity may cause immune rejection, leading to abortion. Immune dysfunction may have a significant role too.

I would like to mention one of our most recent clinical trials in which we have found that NK-1R antagonist aprepitant in combination with dexamethasone may improve the recovery of coronavirus disease 2019 (COVID-19) patients by improving respiratory recovery (Sha et al., 2017). Dexamethasone is already in medical use for strengthening lung functionality in premature deliveries. This is a very strong evidence in support of this current study because it shows that NK-1R is involved in respiratory physiology. It was observed that more adults were infected with COVID-19 as compared to infants and children. It is also in line with our hypothesis and findings. As we discussed earlier that SP/NK-1R may be less expressed in the adult neuromodulatory system, but if it is enhanced in case of nociception, e.g., COVID-19 or another infection, it may lead to a decrease in respiratory function. But in infants, the SP/NK-1R expression is already enhanced due to increased respiratory needs, hence, an activation of the NK-1R system due to stress or nociception may not have an adverse affect.

Human immunoglobulin (IG) has been used widely for the treatment of abortion (Muyayalo et al., 2018). It may modulate the immune mechanisms (Guo et al., 2020) in such a way that it may tolerate the embryo. The immune dysfunction may be enhanced by IG + hCG drug therapy and, hence, improve pregnancy outcomes. It needs to be further explored [45]. In a study, the outcomes were improved by 60% after the treatment. IG + hCG may be suggested to increase the rate of successful pregnancy by modulation of the immune function [45]. The limitations of the study include its small sample size and absence of a control group, which is impossible and immoral due to ethical reasons. As it was not possible to have a control tissue, we used placental tissue and have compared the results with our previous studies. Here, we suggest NK-1R antagonist in addition to the IG + hCG to diagnose and treat spontaneous abortion.

## Data Availability Statement

All datasets presented in this study are included in the article/supplementary material.

## Ethics Statement

The studies involving human participants were reviewed and approved by the University of Lahore Ethics review board. The patients/participants provided their written informed consent to participate in this study.

## Author Contributions

All authors have contributed to the study in writing, planning, immunohistochemistry, microscopy and final approval.

## Conflict of Interest

The authors declare that the research was conducted in the absence of any commercial or financial relationships that could be construed as a potential conflict of interest.
